# Supplementary material for: Complexome profiling of the Chlamydomonas psb28 mutant reveals TEF5 as an early PSII assembly factor
Source: Plant Cell. 2025 Mar 17;37(6):koaf055. doi: 10.1093/plcell/koaf055 (PMC12164590; doi:10.1093/plcell/koaf055)
Supplement: koaf055_Supplementary_Data [file koaf055_supplementary_data.zip › psb28_interactive.zip!/plots/intensity/qualityPlots/peptides/FAP353_Cre12.g560150.t1.2.html]

### Cre12.g560150.t1.2

Trivial name: FAP353  
SeqCoverage [median]: 73.90  
Mass: 22.60 kDa  
  
 

| Sequence | MissedCleavage | Mass [Da] | wtIntensity | muIntensity | Score |
| --- | --- | --- | --- | --- | --- |
| AFADEIGIPFLETSAK | 0 | 1707.87 | 10087.08 | 8085.32 | 210.35 |
| AGGPVVRPQEGKPINSK | 2 | 1732.96 | 45808.48 | 73023.24 | 121.03 |
| GAHGIIVVYDVTDQESFNNVK | 0 | 2304.14 | 3578.72 | 5674.68 | 164.57 |
| KVVEYSVAK | 1 | 1021.58 | 2292.09 | 10872.33 | 162.88 |
| LLLIGDSGVGK | 0 | 1070.63 | 11947.33 | 42016.55 | 120.12 |
| LLVGNKSDLTSK | 1 | 1273.72 | 3311.01 | 9620.19 | 99.52 |
| LLVGNKSDLTSKK | 2 | 1401.82 | 0.00 | 896.15 | 65.81 |
| MASQPIPTK | 0 | 971.51 | 36808.67 | 78910.70 | 191.67 |
| MNPEYDYLFK | 0 | 1318.59 | 6965.30 | 0.00 | 81.59 |
| NATNVEQAFMTMAAEIK | 0 | 1867.88 | 3607.29 | 39348.57 | 155.10 |
| NATNVEQAFMTMAAEIKNR | 1 | 2138.02 | 1420.63 | 8039.38 | 76.15 |
| QWLNEIDRYASENVNK | 1 | 1977.95 | 0.00 | 703.41 | 29.49 |
| TVELDGK | 0 | 760.40 | 7769.25 | 27117.64 | 120.52 |
| TVELDGKVIK | 1 | 1100.64 | 2579.69 | 6704.58 | 119.68 |
| VVEYSVAK | 0 | 893.49 | 5415.63 | 39036.67 | 110.31 |
| YASENVNK | 0 | 923.43 | 4463.10 | 4703.46 | 98.52 |
|  |  |  | 146054.3 | 354752.9 |  |
